# Scale-Free Coupled Dynamics in Brain Networks Captured by Bivariate Focus-Based Multifractal Analysis

**DOI:** 10.3389/fphys.2020.615961

**Published:** 2021-02-03

**Authors:** Orestis Stylianou, Frigyes Samuel Racz, Andras Eke, Peter Mukli

**Affiliations:** ^1^Department of Physiology, Semmelweis University, Budapest, Hungary; ^2^Institute of Translational Medicine, Semmelweis University, Budapest, Hungary; ^3^Department of Radiology and Biomedical Imaging, Yale University School of Medicine, New Haven, CT, United States; ^4^Vascular Cognitive Impairment and Neurodegeneration Program, Oklahoma Center for Geroscience and Healthy Brain Aging, Department of Biochemistry and Molecular Biology, University of Oklahoma Health Sciences Center, Oklahoma City, OK, United States

**Keywords:** scale-free, bivariate, multifractal, functional connectivity, network physiology, electroencephalography

## Abstract

While most connectivity studies investigate functional connectivity (FC) in a scale-dependent manner, coupled neural processes may also exhibit broadband dynamics, manifesting as power-law scaling of their measures of interdependence. Here we introduce the bivariate focus-based multifractal (BFMF) analysis as a robust tool for capturing such scale-free relations and use resting-state electroencephalography (EEG) recordings of 12 subjects to demonstrate its performance in reconstructing physiological networks. BFMF was employed to characterize broadband FC between 62 cortical regions in a pairwise manner, with all investigated connections being tested for true bivariate multifractality. EEG channels were also grouped to represent the activity of six resting-state networks (RSNs) in the brain, thus allowing for the analysis of within- and between- RSNs connectivity, separately. Most connections featured true bivariate multifractality, which could be attributed to the genuine scale-free coupling of neural dynamics. Bivariate multifractality showed a characteristic topology over the cortex that was highly concordant among subjects. Long-term autocorrelation was higher in within-RSNs, while the degree of multifractality was generally found stronger in between-RSNs connections. These results offer statistical evidence of the bivariate multifractal nature of functional coupling in the brain and validate BFMF as a robust method to capture such scale-independent coupled dynamics.

## Introduction

Physiological systems are integrated through a series of intricate connections giving rise to networks of dynamically interacting elements. These may emerge at various scales from molecular pathways (Covert, 2006; Prentki et al., 2020) to the brain connectome (Sporns, 2011) and even at the level of the entire organism (Bashan et al., 2012; Bartsch et al., 2015). The universality of this organizing principle gave birth to the field of network physiology (Bashan et al., 2012; Ivanov and Bartsch, 2014; Bartsch et al., 2015; Ivanov et al., 2016), aiming at unfolding the mechanisms through which diverse physiological systems interact. This goal may be achieved through characterizing various aspects of the temporal coupling between such systems and processes. Novel bivariate analytical methods (Bashan et al., 2012; Schulz et al., 2013; Jalili, 2016) kept advancing the research in this field. Even though many of these methodologies have been proven invaluable for the investigation of scale-specific interactions, they largely neglect the plausible broadband nature of the functional coupling itself (i.e. coupling that spans across a wide range of frequencies). This may, however, become relevant, as many biological processes have been shown to express broadband, scale-free dynamics; examples include the variability of heart rate (Ivanov et al., 1999, 2004; Nunes Amaral et al., 2001; Bartsch et al., 2005), spontaneous brain activity (Ivanov et al., 2009; Lin et al., 2020) or gait variability (Bartsch et al., 2007), to name a few. While these biological functions may contain narrowband components that can also be of interest, their broadband dynamics indicate scale-free (or *fractal*) behavior (Eke et al., 2000). Scale-free features may reveal fundamental aspects of complex systems – such as the human organism – that otherwise remain hidden from traditional methods of analysis. The ubiquity of the univariate fractal dynamics in physiological processes warrants the application of bivariate scale-free time series analysis to study the complexity of coupling between such processes.

Among fields where the human organism (or subsystems thereof) is modeled as a network of functionally coupled elements, brain functional connectivity (FC) studies probably gained the most momentum in past decades (Friston et al., 1993; Biswal et al., 1995; Rubinov and Sporns, 2010; Sporns, 2011; Finn et al., 2015; Lowe et al., 2016; Preti et al., 2017). In that, the network theoretical approach has been shown by many studies to be a powerful tool for the analysis of neural activity patterns (Bullmore and Sporns, 2009; Stam, 2014). According to this framework, the investigated brain regions are considered as nodes of the reconstructed network, while its edges represent the statistically estimated functional coupling between these regions (Rubinov and Sporns, 2010). However, a ‘static’ assessment of FC poses a limitation since the strength of functional coupling between neuronal assemblies has been shown to change over time (Chang and Glover, 2010; Hutchison et al., 2013). Therefore, characterizing the temporal organization of brain network topology requires a model that can account for these time-dependent aspects of FC. This led to the introduction of various tools capable of capturing the dynamic characteristics of brain networks (Dimitriadis et al., 2010; Tagliazucchi et al., 2012; Yu et al., 2015; Preti et al., 2017). Additionally, the ubiquitous presence of scale-free dynamics in the resting-state brain (Werner, 2010; Fraiman and Chialvo, 2012), – especially in the electroencephalogram (EEG) (Lutzenberger et al., 1992; Preißl et al., 1997; Gong et al., 2003; Stam and de Bruin, 2004; Racz et al., 2018b) – encouraged the investigation of power-law scaling in time-varying network properties. Utilizing a combination of dynamic graph theoretical analysis and multifractal time series analysis, we recently revealed that both global (Racz et al.,2018a,b) and local (Racz et al., 2019) properties of functional brain networks fluctuate according to a multifractal pattern, which may also be affected in pathological conditions (Racz et al., 2020). However, a different aspect of connectivity dynamics, namely the scale-free nature of the inter-regional coupling itself, remained inaccessible to these approaches, which mainly utilized a sliding window technique. In contrast to the univariate approach, bivariate multifractal methods – such as detrended cross-correlation analysis (Podobnik and Stanley, 2008) or wavelet-based analysis (Abry et al., 2019; Jaffard et al.,2019a,b)– characterize fractal properties of the coupling between dynamic processes; therefore, they would be able to capture these aspects of functional connections. Furthermore, such approaches could be adapted to the graph-theoretical framework of FC analysis, where edge weights in the network would be assigned as the fractal characteristics of the functional coupling between the investigated brain regions. Networks reconstructed by this approach would inherently represent the fluctuating nature of the connections, in contrast to the traditional way of reconstructing dynamic connections by calculating static indices of interdependence in a sliding window approach. Despite this, to date only a handful of studies investigated the scale-free aspects of functional brain connectivity (Achard et al., 2008; Wang and Zhao, 2012; Ciuciu et al., 2014; La Rocca et al., 2021). In this present work, we set out to address this issue by applying multifractal covariance analysis – introduced earlier by Mukli et al. (2018) – for assessing resting-state functional connectivity reconstructed from EEG measurements.

Some precautions must be addressed, however, when assessing the scale-free properties of empirical signals. In the case of univariate multifractal analysis, it is critical to verify that the obtained indices indeed characterize an inherent property of the observed process, and they not only represent noise or numerical instabilities of the analysis itself (Kantelhardt et al., 2002; Kwapień et al., 2005; Grech and Pamuła, 2012; Rak and Grech, 2018). Similar considerations must be made in the case of bivariate multifractal analysis. Therefore, it is indispensable to verify the presence of true bivariate scale-free coupling by carrying out appropriate statistical tests of power-law cross-coherence (Kristoufek, 2014) and cross-correlation (Wendt et al., 2009; Podobnik et al., 2011; Blythe et al., 2016). Although true multifractality can be confirmed with statistical certainty by extending the testing framework applied for univariate analytical tools (Kantelhardt et al., 2002; Clauset et al., 2009; Roux et al., 2009; Racz et al., 2019, 2020), these methods do not provide much insight into the generating mechanism of bivariate multifractality. Depending on the mechanism, bivariate multifractality could be considered as a consequence of independent univariate dynamics (Wendt et al., 2009; Jaffard et al., 2019a). On the other hand, an appropriate testing framework may identify the genuine scale-free nature of the coupling. This type of bivariate multifractality corresponds to an inherent aspect of the relationship between the processes that otherwise remains undetectable to univariate fractal analysis. For this purpose – namely, to confirm the source of bivariate multifractality –, we devise a testing procedure building on previous studies (Wendt et al., 2009; Kristoufek, 2011) that compares the bivariate fractal measures with their univariate equivalents obtained from the investigated time series to reveal their origin.

So far, the majority of bivariate fractal studies has focused on the analysis of financial time series (Podobnik and Stanley, 2008; Oświęcimka et al., 2014; Pal et al., 2014; Kwapień et al., 2015), while only a few studies applied these tools on physiological datasets (Wang and Zhao, 2012; Ciuciu et al., 2014; La Rocca et al., 2021). Moreover, to the best of our knowledge there have been no studies statistically validating the existence of bivariate multifractality in coupled processes in the human brain or body. Here we apply a novel bivariate method – exploiting the focus-based regression scheme of Mukli et al. (2015) – to investigate if functional connectivity, as reconstructed from EEG recordings, may exhibit a coupled multifractal nature. First, we design and perform a series of statistical tests to confirm true scale invariance and multifractality of individual connections. Second, we assess between-subject and within-subject (i.e., regional) variability of bivariate multifractal indices in order to explore the consistency and discriminatory power of the presented method. Third, we explore whether scale-free coupling displays a topology at the level of large-scale functional networks in the brain. By confirming the plausible bivariate multifractal nature of neural interactions, the present study may not only enhance our understanding of how neural activity is organized in time and space but also provide an efficient analytical pipeline for capturing long-term interdependencies of physiological processes even outside the human brain, on the level of the entire organism.

## Materials and Methods

### Data and Participants

The EEG database analyzed in this study was made publicly available by Sockeel et al. (2016) and consisted of recordings from 12 right-handed, healthy participants (aged 26.6 ± 2.1 years, six females). Each recording contained a 5-minute long segment of resting-state, eyes closed neural activity in which the subjects were lying supine and were listening to an audio recording equivalent to the sounds of an MRI system. EEG tracing was carried out using a 62-channel BrainAmp amplifier, in which the electrodes were arranged according to the international 10–10 system. The sampling rate was set to 5 kHz with the ground and reference electrodes placed at Oz and Cz positions, respectively. Electrode impedance was kept under 10 kΩ during the recordings. The original study was approved by the local ethics committee (Comité de Protection des Personnes–Ile-de-France under the number CPP DGS2007-0555), with measurements being carried out in accordance with the Declaration of Helsinki. All participants provided written informed consent before the measurement. For further details on participants and data collection the reader is referred to the original article of Sockeel et al. (2016).

### Preprocessing

All preprocessing was carried out using Matlab (The Mathworks, Natick, MA, United States). The procedure followed steps of the Batch Electroencephalography Automated Preprocessing Platform (Levin et al., 2018), which uses functions of the EEGLAB toolbox (Delorme and Makeig, 2004) along with custom functions and scripts. First, the data was visually inspected; artifact-free segments of length approximately 55 s long were selected and band-pass filtered with lower and upper cut-off frequencies of 0.5 and 250 Hz, respectively. Additional notch filters at 50, 100, and 200 Hz were applied for line noise removal. Subsequently, the signals were downsampled from 5 kHz to 500 Hz. Further artifact removal was performed using the Harvard Automated Processing Pipeline for Electroencephalography (HAPPE) (Gabard-Durnam et al., 2018). HAPPE implements a series of steps, including wavelet-enhanced independent component analysis followed by independent component analysis with Multiple Artifact Rejection Algorithm (Winkler et al., 2011, 2014). Thus, signal components that likely originate from sources other than neural activity, such as eye movements or scalp muscle contractions, were excluded. Finally, the pruned data was re-referenced to the common average reference. Subsequently, the first 2^14^ datapoints (approximately 33 s) were selected from every preprocessed dataset for further analysis.

### Bivariate Focus-Based Multifractal Analysis

The focus-based multifractal (FMF) analysis framework was introduced by Mukli et al. (2015) in order to provide a robust and efficient way of multifractal time series analysis. Originally, FMF was put forward as a univariate method, i.e., to analyze a single time series. The concept of FMF was then extended to the bivariate domain in a later study (Mukli et al., 2018), with the new method termed bivariate focus-based multifractal analysis (BFMF). Such modification (as detailed below) made the analysis of the multifractal aspect of coupled dynamics feasible and robust, and constitutes the main advantage of BFMF over other bivariate multifractal tools.

**FIGURE 1:**
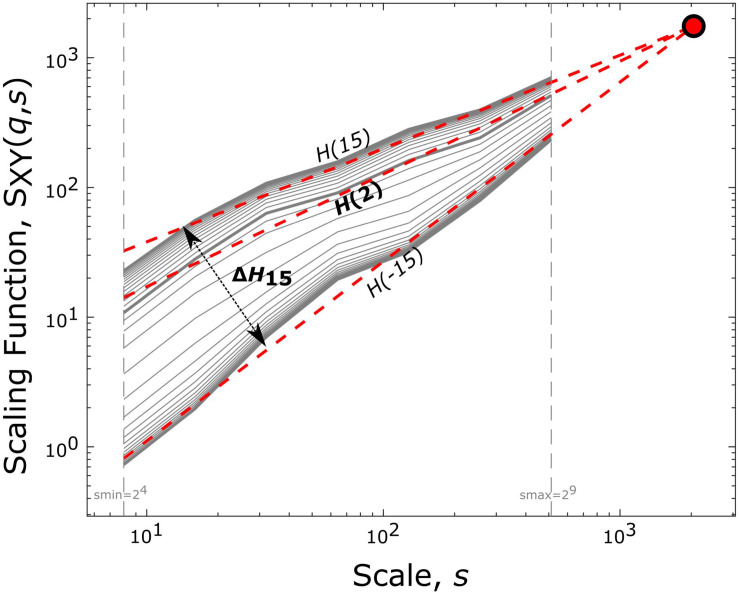
End-point parameters of bivariate focus-based multifractal analysis. Log-log transform of the scaling function [*S*_*XY*_(*q*,*s*)] vs. scale (*s*) relationship is plotted. The generalized Hurst exponent [*H*(*q*)], for several statistical moments (*q*), is acquired via linear regression with the *Focus* (solid red circle) used as a reference point. *H*(2) expresses the long-term correlation between the two time series. At the same time, the degree of multifractality (Δ*H*_15_) is captured by the difference between *H*(*q*) at the minimal (–15) and maximal (15) statistical moments.

Specifically, BFMF is implemented in the time domain using statistical moments (of order *q*) of the scale-wise covariance of sampled time series *X* and *Y* (*cov*_*xy*_) calculated at various window sizes. In that, the scaling function, *S*_*XY*_, is defined according to

(1) $$S_{X\,Y}\,\left(q,s\right)={\left(\frac{1}{N_{s}}\,\sum_{v=1}^{N_{s}}\left|c\,o\,v_{X\,Y}\right|\,{\left(v,s\right)}^{q}\right)}^{1/q}$$

with *N*_*s*_ being the number of non-overlapping windows of size *s* indexed by *v* and *L* = 2^14^ the length of the time series in data points. The cumulatively summed signal is bridge-detrended in each temporal window prior to calculating the covariance. Values of *q* are set to range from −15 to 15 with increments of 1, as this selection of moment orders is sufficient to reliably capture multifractality (Grech and Pamuła, 2012). Scales are defined according to a dyadic scale, i.e., as 2*^*n*^* with *n* ranging from 4 to 9; higher scales were excluded to avoid artifacts due to band-pass filtering. Setting the scale *s* equal to the total signal length *L* renders the sum in (1) independent of *q*. Consequently, in the limit of *s* = *L*, values of *S*(*q*,*s*) converge to one point termed the *Focus* (Figure 1). The *Focus* serves as an iterated reference point in the regression model – based on the equations (18–21) of Mukli et al. (2015) – that simultaneously estimates the best-fitting linear function of log(*s*) to obtain log(*S*(*q*, *s*)) for all values of *q*. The fitting procedure yields a set of power-law exponents (i.e., the slopes of the fitted linear functions), the generalized Hurst exponent function (Barunik and Kristoufek, 2010):

(2) $$S_{X\,Y}\,\left(q,s\right)\propto s^{H\,\left(q\right)}$$

From the estimated *H*(*q*), the ones of particular interest in this study are *H*(2), *H*(−15), and *H*(15). *H*(2) is a measure of global long-term interdependence between *X* and *Y* with the particular case of *H*(2) = 0.5, indicating uncoupled dynamics. *H*(2) < 0.5 shows long-term anticorrelation while *H*(2) > 0.5 positive long-term correlation of the two processes. Since multifractality refers to the temporally altering nature of long-term (cross-) correlations, the degree (or strength) of multifractality can be considered as to what extent this property might change in the process. Since positive and negative moment orders emphasize the contributions of large and small covariance, respectively, a measure characterizing the degree of multifractality can be obtained by calculating the difference between the scaling exponent obtained at the minimal and maximal moments (Grech and Pamuła, 2012; Mukli et al., 2015). Therefore, in our study multifractal strength was captured in Δ*H*_15_ = *H*(-15) – *H*(15), which provides a good and robust approximation of the theoretical limit $\lim_{q\to \infty }H\,\left(-q\right)-H\,\left(q\right)$ (Grech and Pamuła, 2012; Mukli et al., 2015).

### Assessing Multifractality

In order to verify the true multifractal nature^1^ of the functional connections, an array of tests was utilized. The purpose of these tests was to differentiate the true, time-varying scale-free nature of these connections, emerging from the presence of long-term cross-correlations, from those appearing as spurious multifractality (Kantelhardt et al., 2002). First, we tested the power-law dependence of the cross-spectral power on the scale, based on the work of Clauset et al. (2009). In the case of a fractal process, the spectral index (β) of its power spectrum represents the slope of the fitted linear regression of the logarithmic amplitude vs. frequency plot and is proportional to its univariate Hurst exponent, *H*_*univ*_(2) [β = 2*H*_*univ*_(2)-1] (Eke et al., 2002). This relationship also holds in the bivariate case, as the spectral index of the cross-power spectrum of two processes expressing fractal coupling is equivalent to β = 2*H*_*biv*_(2) −1 (Kristoufek, 2014), where *H*_*biv*_(2) is the bivariate Hurst exponent. Therefore, the cross-power spectrum of the two processes is suitable for identifying the plausible power-law dependence in their coupling. For each pair of time series, 40 surrogates were generated whose value of *H*_*univ*_(2) was equal to that of *H*_*biv*_(2), according to the spectral synthesis method (Saupe, 1988). Then, a linear regression model was fitted to the log-log transformed power-spectrum and a Kolmogorov distance was calculated for every generated time series denoting its maximal distance from its power-spectrum (*D*_*univ*_). The distribution of *D*_*univ*_ was compared with the maximal distance of the linear function fitted to the log-log transformed cross-power spectrum of the original connection (*D*_*biv*_). The original connection was considered scale-free (successful test), if

(3) $$D_{b\,i\,v}<\mu \,\left(D_{u\,n\,i\,v}\right)+2\,\sigma \,\left(D_{u\,n\,i\,v}\right)$$

where μ(*D*_*univ*_) and σ(*D*_*univ*_) are mean and standard deviation obtained from the *D*_*univ*_ distribution. Onward, μ() represents the mean and σ() indicates the standard deviation of the distribution in question.

In addition, we examined the detrended cross-correlation coefficients (ρ) calculated for each scale by adopting a method proposed by Podobnik et al. (2011):

(4) $$\rho \,\left(s\right)=\frac{S_{X\,Y}^{2}\,\left(2,s\right)}{S_{X}\,\left(2,s\right)\,S_{Y}\,\left(2,s\right)}$$

where *S*_*X*_(2,*s*), *S*_*Y*_(2,*s*), and *S*_*XY*_(2,*s*) are the scaling function values for scales *s* and the 2nd order statistical moment of time series *X*, *Y* and their connection, respectively. We used a stochastic binomial cascade algorithm (Schumann and Kantelhardt, 2011) to generate a population (100 pairs) of multifractal signals with *L*, *H*(2) and Δ*H*_15_ adjusted to the univariate time series concerned. In line with the refinement of Blythe et al. (2016), all coefficients were tested simultaneously for every scale. Thus, the null hypothesis was only rejected if statistical analysis confirmed that the original ρ(*s*) exceeded that of the surrogate population of cross-correlation coefficients for each scale, yielding an overall *p* < 0.05. Accordingly, the individual significance levels were set to (0.05)^1/6^. Connections that passed the test were considered to have genuine long-term interdependence.

To test if the observed multifractality was due to non-linearities, the following phase randomization scheme was applied. Forty surrogates for each time series were generated by: (*i*) Fourier transforming the data of all channels and (*ii*) randomly permutating the phases before inverse Fourier transformation of the spectrum (Prichard and Theiler, 1994). Since the same permutation was carried out to randomize the phases of data from all channels, this procedure destroyed the non-linear interdependencies between the signals while the linear dependencies remained intact. If the original Δ*H*_15_ (Δ*H*_15_,_*orig*_) did not satisfy the inequality

(5) $$\Delta \,H_{15,o\,r\,i\,g}>\mu \,\left(\Delta \,H_{15,s\,u\,r}\right)+2\,\sigma \,\left(\Delta \,H_{15,s\,u\,r}\right)$$

true multifractality due to non-linearity could not be confirmed.

Shuffling of time series is necessary to distinguish between correlation- and distribution-type bivariate multifractality (Wang et al., 2012). Since shuffling destroys all long-term correlations within (Kantelhardt et al., 2002) and between (Louis et al., 2010) the signals, the shuffled time series are expected to show diminished multifractal profile if their bivariate multifractality is due to long-term correlations. Forty shuffled surrogates were generated from every original signal that resulted in a distribution of *H*(2) and Δ*H*_15_ values for every connection. Consequently, the following inequalities between the original and shuffled datasets were investigated:

(6a) $$H_{o\,r\,i\,g}\,\left(2\right)>\mu \,\left(H_{s\,h\,f\,l}\,\left(2\right)\right)+2\,\sigma \,\left(H_{s\,h\,f\,l}\,\left(2\right)\right)\,\ldots \;\,H_{o\,r\,i\,g}\,\left(2\right)<\mu \,\left(H_{s\,h\,f\,l}\,\left(2\right)\right)-2\,\sigma \,\left(H_{s\,h\,f\,l}\,\left(2\right)\right)$$

(6b) $$\Delta \,{\text{H}}_{15,o\,r\,i\,g}>\mu \,\left(\Delta \,{\text{H}}_{15,s\,h\,f\,l}\right)+2\,\sigma \,\left(\Delta \,{\text{H}}_{15,s\,h\,f\,l}\right)$$

If inequalities (6a) and (6b) hold, then the multifractal character of the connection can be attributed to long-term cross-correlations.

The final assessment was the bivariate-univariate Hurst exponent relationship test, which investigated if further information could be retrieved from bivariate multifractal analysis compared to univariate multifractal analysis. Assume two time series *X* and *Y* with *H*_*XY*_(2), *H*_*X*_(2), and *H*_*Y*_(2) being their bivariate and univariate Hurst exponents, respectively. If *H*_*XY*_(2) does not differ significantly from the arithmetic mean of *H*_*X*_(2) and *H*_*Y*_(2), then the bivariate exponent refers to a scale-free coupling whose Hurst exponent can be predicted from its univariate equivalents (Kristoufek, 2011). In this test, 40 datasets were generated for each time series with the same univariate *H*(2) as that of the original signal, according to the spectral synthesis method (Saupe, 1988). Afterward, the true scale-free nature of the EEG signal was evaluated by performing a univariate power-law test [for details see Racz et al. (2018b)]. For every pair of time series that passed the univariate power-law test, the average of their Hurst exponents, *H*_*XY*,*gen*_(2), was calculated in each of the 40 generated datasets resulting in a distribution. The original *H*_*XY*_(2) was then compared in the following fashion:

(7a) $$H_{X\,Y}\,\left(2\right)>\mu \,\left(H_{X\,Y,g\,e\,n}\,\left(2\right)\right)+2\,\sigma \,\left(H_{X\,Y,g\,e\,n}\,\left(2\right)\right)$$

(7b) $$H_{X\,Y}\,\left(2\right)<\mu \,\left(H_{X\,Y,g\,e\,n}\,\left(2\right)\right)\,-2\,\sigma \,\left(H_{X\,Y,g\,e\,n}\,\left(2\right)\right)$$

If any of the two inequalities was met, then the pair of time series passed the test and their bivariate multifractality was considered intrinsic to the connection. Conversely, a connection failing the bivariate-univariate test was viewed as a case of extrinsic multifractality. This extrinsic multifractality possibly belongs to a functionally non-significant type of bivariate multifractality due to autocorrelation effects (Kristoufek, 2011; Arbabshirani et al., 2014).

### Brain Parcelation and Graph Construction

**FIGURE 2:**
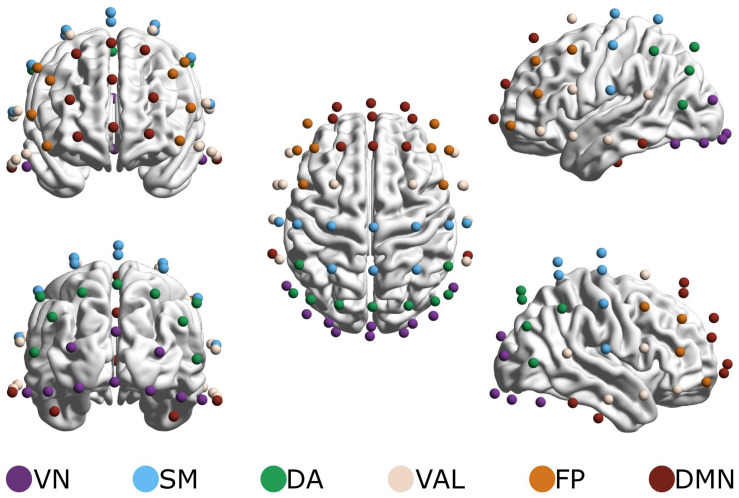
Resting-state networks (RSNs). Electrodes were grouped to represent six RSNs: the visual network (VN, 10 channels), the somatomotor network (SM, 10 channels), the dorsal attention network (DA, 9 channels), the combined ventral attention and limbic networks (VAL, 12 channels), the frontoparietal network (FP, 8 channels) and the default mode network (DMN, 13 channels). Brain maps were created using the BrainNet Viewer software (Xia et al., 2013) after electrode positions were transformed to match a template head using SPM 12b (Penny et al., 2007). The figure originally appeared in Racz et al. (2019).

To reduce the dimensionality of data while also providing a basis for physiological interpretation, a brain parcelation scheme proposed by Giacometti et al. (2014) was applied. The 62 EEG electrodes were grouped based on electrode proximity to seven – functional magnetic imaging (fMRI) labeled – resting-state networks (RSNs) as specified by Thomas Yeo et al. (2011)^2^. Due to the great degree of overlap in electrode locations between the ventral attention and limbic system networks, these were combined into a ventral attention-limbic network (Figure 2), as in Racz et al. (2019). This parcelation thus resulted in 6 RSNs and 15 RSN-to-RSN connections, whose indices were obtained by averaging the obtained values [*H*(2) and Δ*H*_15_] of corresponding connections. We examined connections within each RSN (within-RSNs) and connections between different RSNs (between-RSNs) separately.

### Statistical Analyses

Following the previously described analytical pipeline and brain parcelation scheme, the obtained results were organized into a 12 × 6 within-RSNs matrix (12 subjects, 6 RSNs) and a 12 × 15 between-RSNs matrix (12 subjects, 15 RSN-to-RSN connections) for *H*(2) and Δ*H*_15_, separately. To evaluate the consistency of results among subjects, Kendall’s coefficient of concordance (*W*) was calculated in every matrix. As to verify if cortical localization affected multifractal connection dynamics (i.e., to investigate if multifractal properties of functional connections vary according to various brain regions), we performed the Friedman test with level α*_*s*_* = 0.05 and pairwise comparisons (paired sample *t*-test if distributions were normal, Wilcoxon signed-rank if at least one distribution was non-normal, normality was evaluated by Lilliefors test) followed by Benjamini–Hochberg correction (α*_*s*_* = 0.05) (Yekutieli and Benjamini, 2001).

Finally, to further confirm the significant effect of spatial localization, 100 surrogate datasets were generated, where in every iteration the labels of the channels were randomly permuted before performing the brain parcelation. Subsequently, the Friedman tests were carried out and Kendall’s coefficient of concordance was calculated. The effect of localization was considered statistically significant if the *p*-value obtained from the Friedman test failed to reach significance (i.e., *p* > 0.05) in at least 95 out of 100 cases. *W* values of the original dataset were validated as statistically significant only if they were above the 95th percentile of the *W* resulted from the distribution of the 100 generated datasets.

## Results

### Verifying Bivariate Multifractality

The results of the bivariate multifractality assessment tests are summarized in Table 1. At the subject level, 86.5 ± 5% (mean ± standard deviation) of the total connections passed the power-law test, validating their scale-free nature. The detrended cross-correlation coefficients of all links were found to be significantly higher than those of the surrogate datasets, validating the existence of long-term cross-correlations. All connections passed the phase randomization test, which verified true multifractal coupling due to non-linear interactions. The shuffling test revealed that inequalities (6a) and (6b) held for 99.7 ± 0.3% and 100% of all connections, respectively. These results confirm that the observed multifractality was attributed to long-term cross-correlations.

**TABLE 1:** Success rate of the different scale-free assessing tests at the subject level (mean ± standard deviation).

Performed Test	Success Rate
Power-Law Test	86.5 ± 5%
Detrended Cross-Correlation Coefficient Test	100%
Phase Randomization Test	100%
Shuffling Test – *H*(2)	99.7 ± 0.3%
Shuffling Test – Δ*H*_15_	100%

### Intrinsic vs. Extrinsic Multifractality of Connections

**FIGURE 3:**
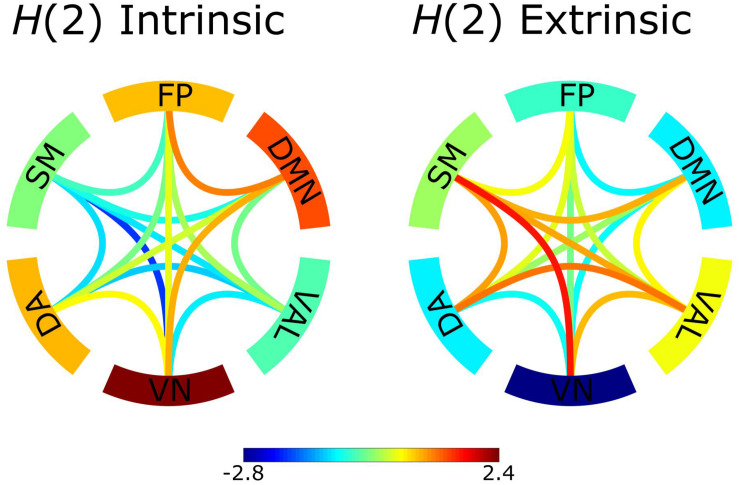
*Z*-scores of intrinsic and extrinsic *H*(2) network connections. The intrinsic network consisted of the *H*(2) values of connections that passed the bivariate-univariate Hurst exponent relationship test, connections that failed were represented as 0. The extrinsic network consisted of the *H*(2) values of connections that failed the bivariate-univariate Hurst exponent relationship test, connections that passed were represented as 0. Subsequently, the *Z*-scores of the connections were calculated. *Z*-scores represent deviation from the population average and their values are indicated by the color bar. The edges serve as the between-RSNs connections with color representing the strength of the connection. The outer ring comprises of the 6 RSNs with the color indicating the *Z*-score of within-RSN connections.

We considered bivariate multifractality as having extrinsic origin if it failed the bivariate-univariate Hurst exponent relation test (equations 7a and 7b) and intrinsic otherwise. The results revealed that a relevant proportion (52.4 ± 6.9%) of the observed functional connections had intrinsic scale-free characteristics. Group-averaged *H*(2) networks separately reconstructed from intrinsic and extrinsic multifractal connections are shown in Figure 3. There is a clear distinction between the two networks [the correlation between the bivariate *H*(2) values consisting of the two networks expressed in Pearson’s *r* = −0.98, *p* < 0.001]. Specifically, within-RSNs connections tend to have stronger intrinsic multifractality, while the between-RSNs links show a higher degree of extrinsic multifractality.

**FIGURE 4:**
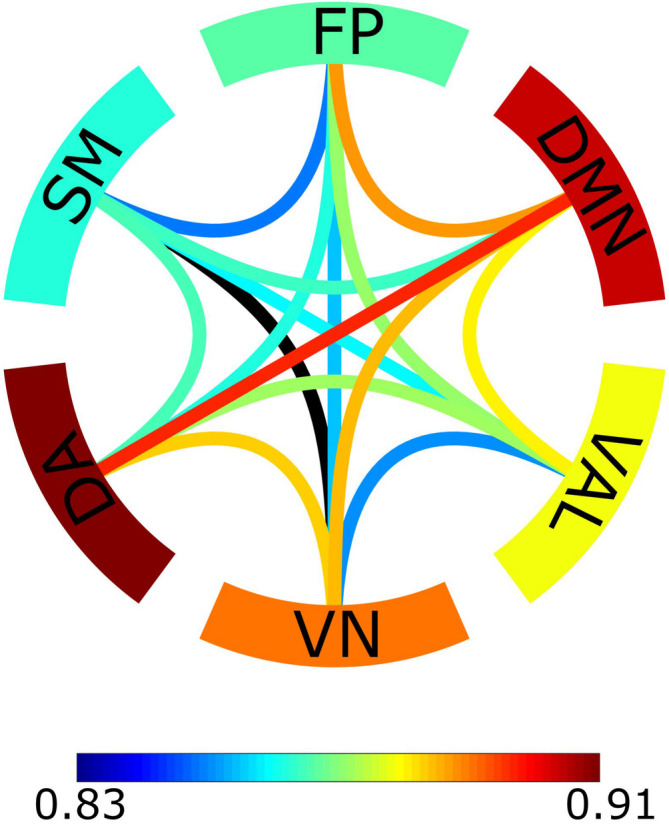
Probabilistic network of intrinsic multifractality. The probability was obtained through the *Z*-score of the original bivariate Hurst exponent of the connection compared to the surrogate distribution created in the bivariate-univariate Hurst exponent relationship test. The edges serve as the between-RSNs connections with color representing the population average probability of the connection showing intrinsic multifractality. The outer ring comprises of the 6 RSNs with the color indicating the population average probability of within-RSNs connections being intrinsically multifractal.

To further illustrate these results, for every connection we calculated its averaged probability of expressing intrinsic multifractality when compared to the distribution of surrogates characterized only by extrinsic multifractality (Figure 4). Two RSNs stood out from the rest, namely the default mode network (DMN) and the dorsal attention network (DA). Not only connections within these RSNs showed a higher probability of intrinsic multifractality when compared to other RSNs, but also the same could be observed for connections linking these to RSNs in comparison to other between-RSNs connections.

### Network Comparison

**FIGURE 5:**
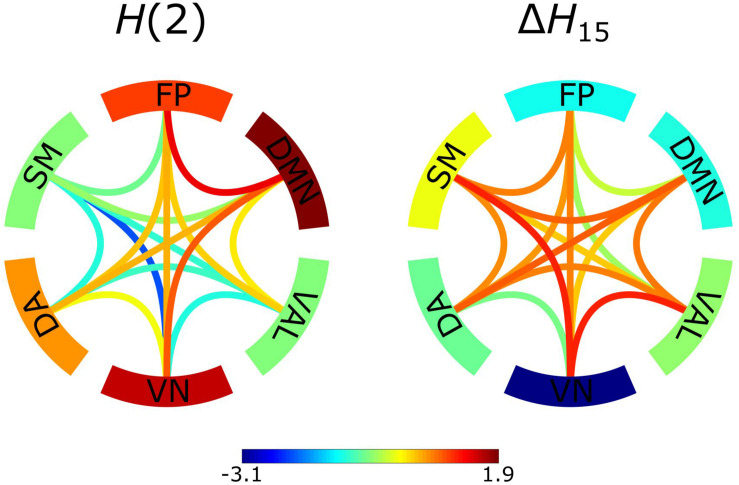
*Z*-scores of constructed networks using *H*(2) and Δ*H*_15_ as functional connectivity estimators. *Z*-scores represent deviation from the population average and their values are indicated by the color bar. The edges serve as the between-RSNs connections with color representing the strength of the connection. The outer ring comprises of the 6 RSNs with the color indicating the population average strength of the within-RSNs connections.

Two networks were constructed from the results obtained by BFMF analysis, one from *H*(2) and one from Δ*H*_15_ values of functional connections (Figure 5). The two networks showed markedly different patterns (the correlation between the two networks expressed in Pearson’s *r* = −0.6609, *p* < 0.01). Specifically, it appeared that *H*(2) and Δ*H*_15_ of functional connections were inversely related, as within-RSNs connections expressing higher *H*(2) values could be characterized with lower Δ*H*_15_, and vice versa. The same inverse relationship could be observed for the multifractal properties of between-RSNs connections, although less prominently.

### Effect of Subject and Regional Variability

**FIGURE 6:**
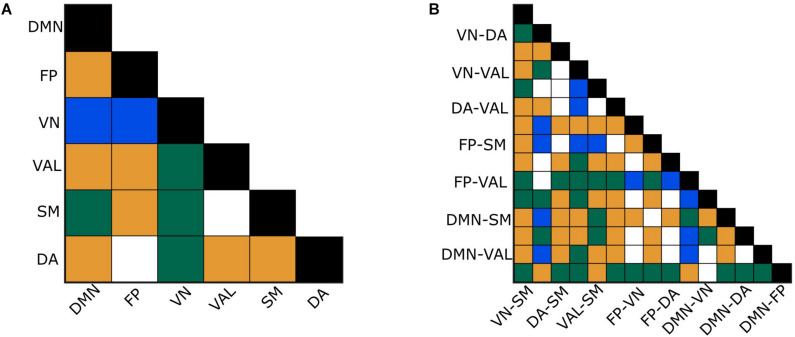
Effect of regional variability. Significance of connection-to-connection comparisons of within- **(A)** and between- **(B)** RSNs after the appropriate correction for *H*(2) and Δ*H*_15_. Blue: Only Δ*H*_15_ comparison test was significant. Orange: Only *H*(2) comparison test was significant. Green: Both *H*(2) and Δ*H*_15_ comparison tests were significant.

The between- and within-subject variability of connections in both network types were analyzed using Kendall’s *W*, Friedman tests and paired difference tests. For the *H*(2) network, Kendall’s *W* values of 0.72 and 0.65 were obtained for between- and within-RSNs connections, respectively, indicating strong concordance among subjects. Friedman tests revealed a significant main effect of localization (*p* < 0.0001). 68.6% of the between-RSNs and 73.3% of the within-RSNs of the pairwise *post hoc* tests were found significant. The *W* values of the Δ*H*_15_ network were 0.44 and 0.47 for between- and within- RSNs connections, suggesting moderate subject agreement. Friedman test again indicated a significant main effect of localization for the Δ*H*_15_ values of functional connections (*p* < 0.0001), while 40% of the paired tests of between- and within-RSNs connections indicated a significant difference. Moreover, the two different networks displayed mostly different connections as statistically different (Figure 6). Table 2 summarizes the results of the statistical tests performed on *H*(2) and Δ*H*_15_ networks.

**TABLE 2:** Results of Kendall’s *W*, success rate for individual paired difference tests after correction and Friedman test for *H*(2) and Δ*H*_15_ for between- and within- RSNs.

	Kendall’s *W*	Paired difference test success rate	Friedman Test *p*
*H*(2) Between-RSNs	0.72	68.6%	<0.0001
*H*(2) Within-RSNs	0.65	73.3%	<0.0001
Δ*H*_15_ Between-RSNs	0.44	40%	<0.0001
Δ*H*_15_ Within-RSNs	0.47	40%	<0.0001

To further validate that cortical localization significantly impacted connection dynamics, the parcelation scheme was evaluated against *n* = 100 spatially shuffled surrogates (see section “Materials and Methods”). In that, only 1% of the generated datasets showed *p*-values smaller than 0.05 after shuffling the channel labels. Moreover, Kendall’s *W* values for between-RSNs and within-RSNs for both *H*(2) and Δ*H*_15_ were found significantly higher than those obtained from randomized data. These results further confirm that functional connections linking various regions of the brain express different scale-free characteristics.

## Discussion

In this study, we present a novel bivariate adaptation of focus-based multifractal time series analysis and show its applicability for studying the spatiotemporal organization of functional brain networks. The main contribution of this work, therefore, lies with the utilization of the BFMF method and its associated statistical framework for the reconstruction of brain networks based on scale-free coupled dynamics. In that, using detrended covariance as a time-domain measure for BFMF, we examined the fractal connectivity by calculating bivariate *H*(2) and Δ*H*_15_ for each pair of processes, thereby assessing linear and non-linear aspects of their scale-free dynamics, respectively. The applied tests were essential in validating our findings and confirming that most of the connections were indeed multifractal. Moreover, with a combined application of bivariate and univariate focus-based multifractal analysis, we revealed whether the observed cross-regional temporal dynamics emerged from genuine scale-free interactions intrinsic to the connection, or were simply a consequence of long-term autocorrelation present in both processes. The reconstructed networks and their topology were highly consistent among subjects, while significant regional variability over the cortex was also observed. Our findings demonstrate that BFMF is an analytical tool capable of capturing scale-free coupled dynamics of physiological networks, a feature that may otherwise remain undetected by univariate fractal analytical methods.

### Bivariate Multifractality in the Brain

Despite the ubiquity of scale-free characteristics in neural dynamics (He et al., 2010), only a limited number of studies investigated the fractal nature of the functional coupling between these processes. Ciuciu et al. (2014) assessed scale-free coupling of neural dynamics from fMRI datasets using frequency- and wavelet-based measures, thereby having to resort to an inherently low temporal sampling rate limiting both the precision and possible interpretation of their results. Other functional connectivity studies verified the presence of scale-free coupling in magnetoencephalography recordings using wavelet coherence function (La Rocca et al., 2021). The only bivariate scale-free study of EEG datasets was an exploratory investigation reporting significant differences in the bivariate multifractal profiles between young and elderly populations (Wang and Zhao, 2012).

Although these works reported on relevant aspects of neural dynamics, they did not provide statistical tests for the validation of the true multifractal nature of the investigated connections. This study aimed to rectify this limitation by adapting univariate scale-free assessment tests in the bivariate setting, as well as improving already-existing bivariate equivalents. Most of the analyzed connections in our study showed genuine multifractal coupling due to long-range cross-correlations, as indicated by the high success rates in the power-law, detrended cross-correlation, phase randomization and shuffling tests. It was indispensable to examine the presence of power-law relationship since coupled oscillatory dynamics confined to a specific time scale/frequency range might be present in our dataset. Robust detection of this feature was ensured by a statistical framework implemented in the frequency domain (Clauset et al., 2009). Moreover, the detrended cross-correlation coefficients of the original connections were significantly different from those of surrogate data at every scale, directly indicating the presence of scale-free long-term cross-correlations in the time domain (Podobnik et al., 2011). The purpose of phase randomization was to yield a population of surrogate data with abolished non-linearity (Prichard and Theiler, 1994). Comparing the multifractal characteristics of the surrogate population with those of the original data revealed that multifractality was indeed a consequence of the non-linear nature of the coupling between processes. The shuffling test, which distinguished between correlation- and distribution-type multifractality (Kantelhardt et al., 2002), indicated that most of our connections were of the former type. However, the bivariate multifractality of EEG-signals observed in this study can be attributed only partly to long-term cross-correlations, since the finite size effect will always contribute to the observed multifractality (Grech and Pamuła, 2012). To the best of our knowledge, our study is the first to statistically validate the existence of multifractality between elements of a physiological network, in this case the brain. However, our findings may also open the way for the investigation of other networks of the human organism, whose constituents also express scale-free dynamics [such as heart rate variability (Ivanov et al., 1999, 2004; Nunes Amaral et al., 2001; Bartsch et al., 2005), gait variability (Bartsch et al., 2007), muscle activity (Santuz and Akay, 2020), breathing (Fadel et al., 2004), or blood glucose level fluctuations (Weissman and Binah, 2014)]. By applying BFMF to assess the coupling in such systems, novel aspects of their interactions could be revealed that have not yet been accounted for.

An essential aspect of scale-free interactions is whether the observed multifractality is an intrinsic property of the relationship. Considering the fact that covariance estimation is influenced by the autocorrelation of the signals (Arbabshirani et al., 2014), we can safely assume that the intrinsic multifractality of a connection represents true statistical interdependence between the different brain regions while a large part of extrinsic multifractality could be ascribed to autocorrelation effects (Kristoufek, 2011). According to Figures 3, 4, while the between-RSNs connections showed a *mostly* extrinsic type of multifractality, the within-RSNs connections *mainly* featured intrinsic multifractality. This finding to some extent can be evident since a higher number of intrinsic (i.e., true) multifractal connections could be expected to exist within functionally cohesive neural populations, such as RSNs (van den Heuvel et al., 2010), as opposed to the links between them. These results may further support the notion that cortical regions that are considered to form RSNs are: (*i*) indeed functionally coupled and (*ii*) segregated from the rest of the brain (to some extent). Another noteworthy finding illustrated by Figure 4 is that the default mode network, dorsal attention network and the connections between them showed the highest probability of intrinsic multifractality. DMN comprises of brain regions with increased FC during idling (Chen et al., 2008), and considering that the analyzed datasets were obtained in the resting-state, we can expect strong within-DMN connectivity. On the other hand, DA has increased FC during tasks that require attention (Vossel et al., 2014), making the high probability of intrinsic multifractality of connections both within DA and between DA and DMN unexpected. A recent study (Murphy et al., 2020) indicated an indirect functional connection between DMN and DA mediated by the frontoparietal network, providing partial support for our findings of a high chance of intrinsic multifractality in the DMN-DA connections. Although our parcelation scheme prevents us from drawing stronger conclusions on the activities of RSNs, our findings still allow a clear demonstration of the regional variability of scale-free coupling in large-scale brain networks.

The origin of scale-free/multifractal nature in brain activity is still an active field of research, which yet remains to be fully resolved. One plausible explanation may be provided from the study of critical systems. Accordingly, the brain can be considered as a complex system that exists at the brink of order and chaos (Weil, 1994; Beggs and Timme, 2012; Hesse and Gross, 2014), with its fine-tuned equilibrium and 1/*f*-dynamics indicating the presence of self-organized criticality (SOC) (Bak et al., 1987; Buzsáki, 2006). The concept of SOC emphasizes that the brain tends to operate in a critical state (Bonachela et al., 2010; Hesse and Gross, 2014), where even a local perturbation can elicit a global response. In SOC-based interpretations of neural dynamics, criticality is achieved by fine-tuning a control parameter inherent to the brain. Despite options emerging from electrophysiological experiments (Freeman, 2004; Buzsáki, 2006), the identity of this control parameter remains elusive, sustaining a dispute within the neuroscience community over the relevance of SOC in explaining the observed dynamics (Beggs and Timme, 2012; Hesse and Gross, 2014). A likely candidate is a balance between incoming excitatory and inhibitory signaling of the neuronal populations. It has already been demonstrated that power-law scaling at local field potentials and global electromagnetic brain signals (Beggs and Timme, 2012; Poil et al., 2012) can emerge through such equilibrium of incoming excitatory and inhibitory stimuli. A similar model, attributed to the balance between the two divisions of the autonomic nervous system, has been suggested as the source of the scale-free fluctuations of the heart rate variability (Ivanov et al., 1998; Nunes Amaral et al., 2001). In line with these considerations, the stochastic influx of excitatory/inhibitory signals may be a possible source of bivariate multifractality of the brain networks, however this hypothesis requires further research.

### Aspects of Functional Coupling Captured by BFMF

In this study, BFMF was used as a functional connectivity estimator, from which two brain networks were reconstructed. A network was defined by assigning the bivariate *H*(2) values as edge weights, reflecting the topology of long-term cross-correlation. Similarly, bivariate Δ*H*_15_ values were assigned to all connections forming a network that displays the topology of the multifractal strength. It should be emphasized that the obtained scale-free pattern of functional connections appeared highly consistent among subjects, in agreement with previous studies (Gong et al., 2003). Moreover, our results indicated significant regional variability for both within- and between- RSNs connections. This regional variation was notably different between the *H*(2) and Δ*H*_15_ networks (Figure 6), emphasizing that these two measures of scale-free dynamics are complementary to each other also in the bivariate setting. The complementary nature of *H*(2) and Δ*H*_15_ has already been demonstrated in the univariate fractal analysis (Mukli et al., 2015; Racz et al., 2018b). Furthermore, the two networks yielded opposite patterns regarding their topologies, i.e., those connections with high *H*(2) values were found to express low Δ*H*_15_ values and vice versa (Figure 5). A similar relationship between univariate *H*(2) and Δ*H*_15_ was found in an earlier study; however, only for delta band connections (Racz et al., 2018b). In that work, synchronization likelihood was used as a dynamic functional connectivity estimator and multifractal properties of time-varying synchronization levels (i.e., dynamic functional connections) were estimated using the univariate FMF method. Since three out of the six scales (128, 256, 512 data points) used in the current analysis fall within the delta band (0.5–4 Hz), this may explain the observed similarities with the study discussed above.

A source of inconsistency among FC studies may emerge from the application of various thresholding schemes. In that, most studies use some form of pruning procedure to exclude connections from the reconstructed networks that may be spurious or originating from noise (Rubinov and Sporns, 2010; van den Heuvel et al., 2017). Given that the primary goal of the study was to demonstrate the existence of multifractal coupling in brain networks as well as the introduction of a new method for its assessment, our main analytical pipeline did not contain a thresholding step. Nevertheless, in order to explore the plausible effect of thresholding on scale-free network topology we applied a parallel pipeline, which included thresholding as follows. The Δ*H*_15_ networks only included connections that passed all four multifractality assessment tests. *H*(2) networks consisted of links that successfully passed the power-law, detrended-cross correlation and shuffling tests. Further details about this parallel analysis are provided in the Supplementary Material. Notably, the localization of intrinsic multifractality and the *H*(2) and Δ*H*_15_ networks architectures were highly similar to the unthresholded case, while the regional variability and subject concordance was found diminished (Supplementary Figures S1–S3 and Supplementary Table S1). The inference of this comparison is that intrinsic multifractality only marginally depends on the thresholding procedures while between- and within-subject variability of *H*(2) and Δ*H*_15_ networks is clearly influenced.

### Comparison of BFMF With Scale-Dependent FC Estimators

Given the novelty of our method, it is important to compare our results to those obtained by other FC methods commonly used in the literature (van den Heuvel and Fornito, 2014). For this purpose, we also reconstructed brain networks with the aid of Pearson correlation (*r*) and Mutual Information (MI) (details found in Supplementary Material). The purpose of this testing was to investigate if BFMF could reveal network architectures different from those obtained with scale-dependent linear or non-linear methods, thus implying its utility in capturing novel aspects of spatio-temporal neural dynamics. Since *r* and MI are indeed scale-dependent, we analyzed our signals at the same six scales as in BFMF analysis (16, 32, 64, 128, 256, and 512 data points) in a non-overlapping windowed manner. While the *r* networks showed a similar distribution of FC as the *H*(2) network (Figure 5 and Supplementary Figure S4), the MI networks did not resemble any of the two BFMF networks (Figure 5 and Supplementary Figure S5). Moreover, regional variability was more significant in the *r* and MI networks (Supplementary Table S2), suggesting the influence of oscillatory dynamics. These oscillatory dynamics, despite their physiological correlates, cannot capture the scale-independent network connectivity evaluated by BFMF. To conclude, these results call for the careful interpretation of observed functional connectivity patterns pertinent to the estimator used for their assessment, while also highlight the fact that BFMF captured patterns of neural dynamics that remained undetected by *r* or MI.

### Limitations and Future Perspectives

Finally, the limitations of this study should also be addressed. The 5-minute eyes-closed resting-state EEG recordings did not allow for a comparison of networks under different mental states, which have been shown to influence the fractal properties of neural dynamics (Ciuciu, 2012; Ciuciu et al., 2014). Nevertheless, as the primary objective of this study was to demonstrate the applicability of BFMF as a novel tool for reconstructing physiological networks of functional significance. For that purpose, a homogenous resting-state EEG dataset was sufficient, while subsequent research should indeed consider more elaborate experimental paradigms. Even though more than half of the connections showed intrinsic multifractality in every subject, at the population level there was only a tendency (maximal probability was 0.91) of localization of intrinsically multifractal connections within the resting-state networks (Figure 4). A possible explanation of this could be the low sample size of the study. It is reasonable to assume that future studies with a larger subject cohort could further confirm enhances the significance of this dichotomous model. Due to limitations of the applied parcelation scheme in demonstrating RSN-dependent contrast of bivariate multifractal measures, more elaborate experimental paradigms are needed for a thorough investigation of the origin of the scale-free character between and within the different RSNs via source-reconstruction (La Rocca et al., 2021). Infra-slow neural activity (<0.5 Hz) was not considered in this study since our preliminary investigations showed that breakpoints of the scaling function appear around 0.5 Hz (for further details in bimodal multifractal analysis, see Nagy et al., 2017). In future investigations, low-frequency EEG could be examined by a scaling-range adaptive, bimodal extension of BFMF, which appears as a reasonable next step considering recent advances in the analysis of multimodal fractal time series (Nagy et al., 2017; Mukli et al., 2018). These investigations should include high-pass filtering with a much lower cut-off frequency, which however will also require appropriate measurement length and sampling rate. The relevance of this consideration is supported by findings from fMRI recordings indicating that frequencies closer to 0.01 Hz contribute to multifractal functional connections to a greater extent (Ciuciu et al., 2014). Our study investigated only one exemplary case of physiological networks, namely functional networks of the human brain. In general, investigation of any biological process observed for a sufficiently long period of time and sampled at adequate temporal resolution could benefit from this method, as the BFMF framework enriches the analytical repertoire suitable for investigating dynamic physiological networks. In fact, multifractal covariance analysis has revealed a genuine scale-free coupling between oxy- and deoxyhemoglobin fluctuations (Mukli et al., 2018) that could be ascribed to mechanisms of neurovascular coupling. A certainly important direction of further research should be to implement this methodology in clinical studies, especially in psychiatry, where new biomarkers with good performance and reliability in individualized treatment are much needed (Topol, 2019). Finally, even though BFMF was developed for the study of physiological networks, it can still be applied in a variety of other disciplines, like in the field of economics on which the bivariate multifractal analysis has been focusing so far (Oświęcimka et al., 2014; Pal et al., 2014).

## Conclusion

Here we introduced the bivariate focus-based multifractal analysis for the dynamic investigation of physiological networks and showed that it captures novel features of resting-state brain network dynamics. Namely, supported by statistical testing, BFMF could reveal true multifractality in most of the functional connections estimated from EEG signals. Moreover, topological patterns identified with BFMF appeared robust, as indicated by high subject concordance and strong regional variability. Our results could facilitate further research on brain networks under different experimental conditions using bivariate multifractal analysis, as well as on extended physiological networks at the level of the entire organism.

## Data Availability Statement

The original contributions presented in the study are included in the article/Supplementary Material, further inquiries can be directed to the corresponding author/s.

## Ethics Statement

The studies involving human participants were reviewed and approved by Comité de Protection des Personnes–Ile-de-France. The patients/participants provided their written informed consent to participate in this study.

## Author Contributions

OS implemented the analytical framework, performed the data analysis and interpretation, and wrote the first draft of the manuscript. FR contributed to data visualization, data analysis and manuscript development. AE provided the conceptual guidance and supervision throughout the study. PM developed the code for BFMF and specified the concept and aims of the study. All the authors contributed to reviewing the manuscript and approved its final version.

## Conflict of Interest

The authors declare that the research was conducted in the absence of any commercial or financial relationships that could be construed as a potential conflict of interest.

## Footnotes

1 The multifractal nature of the coupling was based on the dichotomus model of fractional Gaussian noise and fractional Brownian motion (Eke et al., 2000) extended to the multivariate setting (Lavancier et al., 2009).

2 Note that the optimal method of matching EEG channels to RSNs (or more correctly to regions of interest) is by source-reconstruction (Michel and Brunet, 2019) and subsequent generation of time-series for each RSN. Our parcelation targeted mainly the dimensionality reduction and hence no strong conclusions about RSNs should be made based on this. For a source-reconstructed scale-free functional connectivity study we suggest reading (La Rocca et al., 2021).
