# Supplementary material for: Scale-Free Coupled Dynamics in Brain Networks Captured by Bivariate Focus-Based Multifractal Analysis
Source: Front Physiol. 2021 Feb 3;11:615961. doi: 10.3389/fphys.2020.615961 (PMC7887319; doi:10.3389/fphys.2020.615961)
Supplement: Supplementary file 1 [file Data_Sheet_1.PDF]

Scale-free Coupled Dynamics in Brain Networks Captured by Bivariate Focus-Based Multifractal Analysis - Supplementary Material

1. Thresholding of BFMF Networks

Each connectivity matrix was thresholded in order to exclude spurious connections. The ΔH_{15} network consisted only of connections that passed all four multifractality tests. The $H(2)$ network included links that passed the power-law, detrended cross-correlation and $H(2)$ part of the shuffling tests. The phase-randomization and ΔH_{15} part of shuffling tests were not taken into consideration for the thresholding of $H(2)$ networks because some of the connections could show monofractal (i.e. successful power-law, detrended cross-correlation and $H(2)$ part of the shuffling tests) but not multifractal (negative phase-randomization and ΔH_{15} part of shuffling tests) character. Moreover, the applied thresholding scheme did not consider the dichotomous model of extrinsic/intrinsic multifractality. The reason is that even though the majority of extrinsic multifractality is due to autocorrelation effects, a part of it can still be due to interdependence between the different brain regions. The connectivity matrices yielded graphs in which the weight of the rejected edges was set to 0.

The analysis showed that a large part (59.0±5.9%) of the observed functional connections had intrinsic scale-free characteristics. **Figure S1** illustrates two $H(2)$ networks, each responsible for either the intrinsic or extrinsic multifractal connections. There is a clear distinction between the two networks (the correlation between the two networks expressed in Pearson's r =-0.95, $p<0.001$). The within-RSNs connections tend to have stronger intrinsic multifractality, while the between-RSNs links show a higher degree of extrinsic multifractality. Despite the statistically significant anticorrelation between the two networks and the more than 50% intrinsically multifractal connections per subject, the probability of a connection being intrinsically multifractal did not reach significance at a population level (**Figure S2**). Nevertheless, the same high probability of intrinsic multifractality within-DMN, within-DA and between DMN-DA connections is observed, as in unthresholded networks (**Figure 4**).

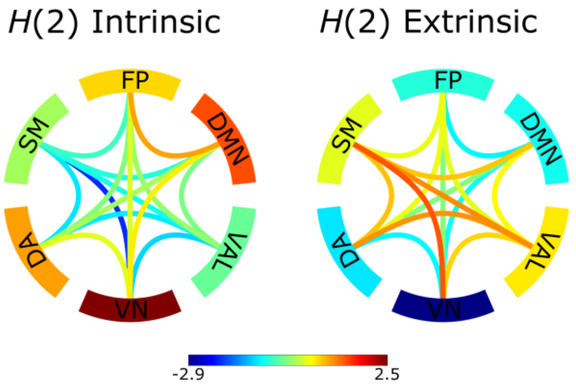

Figure S1. *Z-scores of intrinsic and extrinsic thresholded H(2) network connections*. The intrinsic network consisted of the $H(2)$ values of connections that passed the bivariate-univariate Hurst exponent relationship test, connections that failed were represented as 0. The extrinsic network consisted of the $H(2)$ values of connections that failed the bivariate-univariate Hurst exponent relationship test, connections that passed were represented as 0. Subsequently, the Z-scores of the connections were calculated. *Z*-scores represent deviation from the population average and their values are indicated by the color bar. The edges serve as the between-RSNs connections with color representing the strength of the connection. The outer ring comprises of the 6 RSNs with the color indicating the *Z*-score of within-RSN connections.

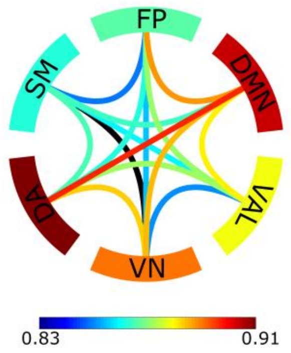

Figure S2. *Probabilistic network of intrinsic multifractality after thresholding*. The probability was obtained through the Z-score of the original bivariate Hurst exponent of the connection compared to the surrogate distribution created in the bivariate-univariate Hurst exponent relationship test. The edges serve as the between-RSNs connections with color representing the population average probability of the connection showing intrinsic multifractality. The outer ring comprises of the 6 RSNs with the color indicating the population average probability of within-RSNs connections being intrinsically multifractal.

The two thresholded networks showed statistically significant different patterns (the correlation between the two networks expressed in Pearson's $r = -0.73$, $p<0.01$) (**Figure S3**). The $H(2)$ network showed higher values for within-RSNs connections when compared to the ΔH_{15} network. The contrary was observed in the between-RSNs connectivity, with that of ΔH_{15} network being stronger, although in less extent.

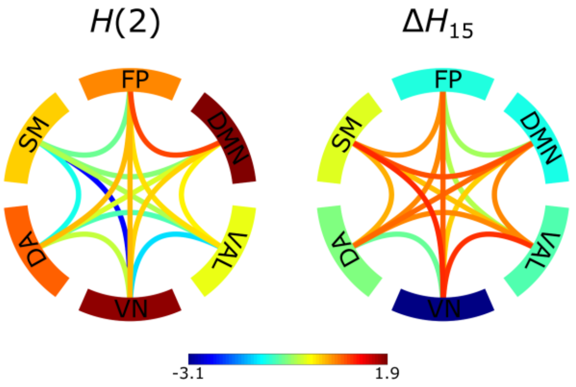

Figure S3. *Z-scores of constructed networks using thresholded H(2) and ΔH*$_{15}$ *as functional connectivity estimators. Z*-scores represent deviation from the population average and their values are indicated by the color bar. The edges serve as the between-RSNs connections with color representing the strength of the connection. The outer ring comprises of the 6 RSNs with the color indicating the population average strength of the within-RSNs connections.

Finally, **Table S1** summarizes the statistical tests applied in the thresholded networks. For the *H*(2) network, the between- and within- RSNs *W* values of 0.50 and 0.35 were acquired respectively, indicating moderate concordance among subjects. Friedman tests revealed a significant main effect of localization (p<0.0001). 40% of the between-RSNs and 33.3% of the within-RSNs of the pairwise post hoc comparisons appeared significant. The *W* values of the ΔH_{15} network were 0.37 and 0.41 for between- and within- RSNs connections, suggesting moderate subject agreement. The Friedman test indicated a significant main effect of localization for the ΔH_{15} values of functional connections (p<0.0001), while 26.7% of the between-RSNs and within-RSNs paired comparisons were successful.

Table S1. Results of Kendall's W, success rate for individual paired comparisons after correction and Friedman test for *thresholded* H(2) and ΔH_{15} for between- and within- RSNs.

		**Kendall's *W***	**Paired difference test success rate**	**Friedman Test *p***
***H*(2)**	between-RSNs	0.50	40%	<0.0001
	within-RSNs	0.35	33.3%	
**ΔH_{15}**	ΔH_{15} between-RSNs	0.37	26.7%	
	ΔH_{15}within-RSNs	0.41	26.7%	

2. Pearson Correlation and Mutual Information Analysis

To verify if BFMF captures novel aspects of brain functional connectivity, we generated reference networks using two widely used estimators of functional connectivity, Pearson correlation (*r*) and Mutual Information (MI) (van den Heuvel and Fornito, 2014).

Pearson correlation is a widely used measure of FC and *per se* it can identify linear interdependence between two processes. The Pearson correlation coefficients of all possible channel pairs were calculated using built-in MATLAB functions. Similarly to BFMF analysis, *r* was evaluated in non-overlapping windows of size *s*, with values of *s* set equal to those used in BFMF. The signal was bridge-detrended in each temporal window before calculating *r*. Finally, the mean *r* was calculated for every *s*.

Mutual Information is another frequently used measure of FC that captures both linear and nonlinear dependencies (Shannon, 1948; Steuer et al., 2002). This is achieved by measuring the deviation of the estimated joint probability distribution of two variables, in our case neurophysiological signals, from a theoretical joint probability distribution assuming independence. If those two joint probability distributions correspond (i.e. the two time series are indeed statistically independent), then the value of MI between these time series is 0. Positive values of MI represent statistically dependent time series with higher values corresponding to more substantial interdependence (Steuer et al., 2002). Since MI identifies the interdependence of two processes based on their empirical probability distribution, it is considered a model-free measure, unlike estimators obtained by BFMF analysis. Despite the theoretically simple calculation of MI, this is a computationally-intensive process with the constant emergence of new innovative algorithms (Jiang et al., 2010). In this study, we implemented the second algorithm proposed in (Jiang et al. 2010) for estimating zero-lag Mutual Information. Pairwise calculation of MI from the bridge-detrended EEG datasets was performed according to the same non-overlapping windowing scheme and time scales as in Pearson correlation analysis.

The Pearson- and MI-derived networks were investigated with the same statistical pipeline as the BFMF-derived networks. In that, we calculated Kendall’s coefficient of concordance (*W*), *p*-value of Friedman test and post-hoc paired tests (paired sample t-test if distributions were normal, Wilcoxon signed-rank if at least one distribution was non-normal, normality was evaluated by Lilliefors test) corrected by Benjamini-Hochberg correction (Yekutieli and Benjamini, 2001).

As seen in **Figure S4**, there is a scale-independence between the scales 64, 128, 256 and 512 in the Pearson networks. The results agree with Bassett et al. (Bassett et al., 2006), where a frequency-independence for the mean degree, clustering coefficient and minimum path length in brain networks was found. On the other hand, scales 16 and 32 did not show that scale-independence, possibly because they represent higher (beta and gamma) EEG bands, which are not prominent during eyes-closed resting-state. As to the Mutual Information analysis, each scale showed different network architecture, except scales 128 and 256 (**Figure S5**). This polymorphism in MI networks validates our belief that the commonly used functional connectivity estimators are prone to *a priori* assumptions. Both *r* and MI networks demonstrated higher subject concordance and regional variability compared to the BFMF networks, indicating the influence of oscillatory dynamics. **Table S2** summarizes the statistical results of our analysis of *r* and MI networks.

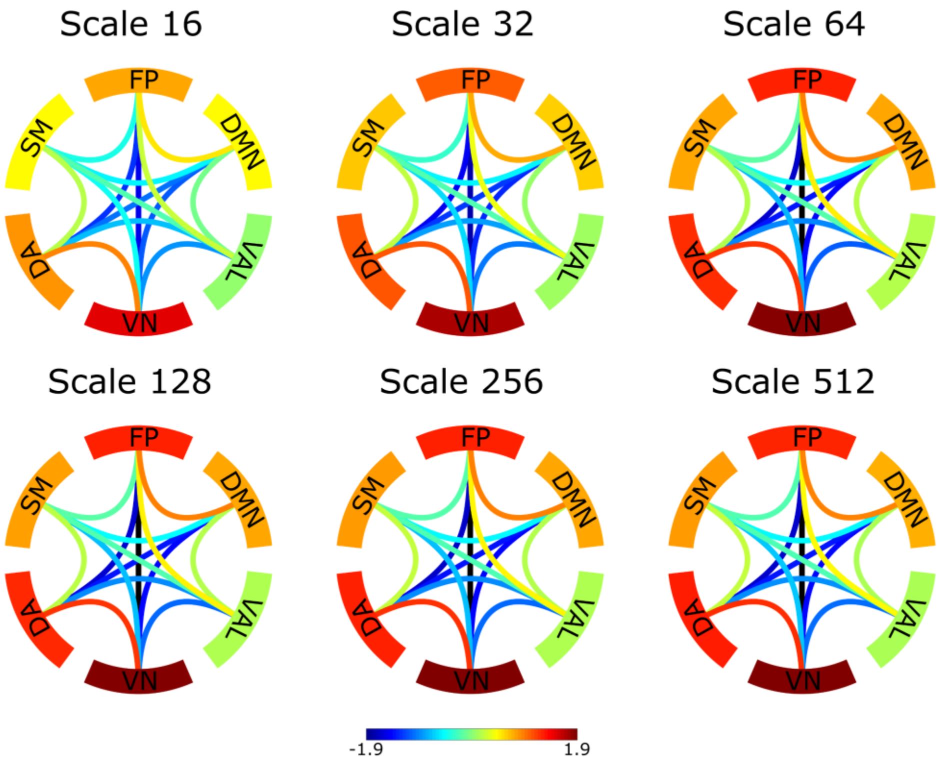

Figure S4. *Z-scores of constructed networks using Pearson correlation as functional connectivity estimators for six different scales*. The edges serve as the between-RSNs connections with color representing the population average strength of the connection. The outer ring comprises of the 6 RSNs with the color indicating the population average strength of the within-RSNs connections.

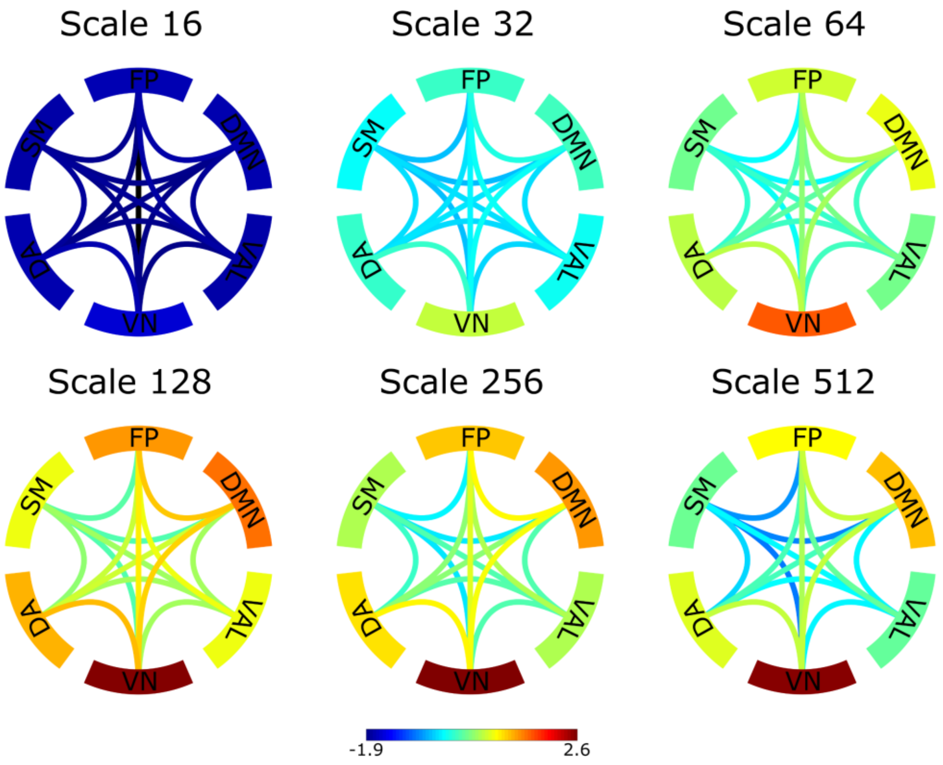

Figure S5. *Z-scores of constructed networks using Mutual Information as functional connectivity estimators for six different scales.* The edges serve as the between-RSNs connections with color representing the population average strength of the connection. The outer ring comprises of the 6 RSNs with the color indicating the population average strength of the within-RSNs connections.

Table S2. Results of Kendall's W, success rate for individual paired comparisons after correction and Friedman test for Pearson correlation (r) and Mutual Information (MI) for between- and within-RSNs.

		**Kendall's *W***	**Paired difference test success rate**	**Friedman Test *p***
**Scale 16**	*r* between-RSNs	0.96	94.3%	< 0.0001
	r within-RSNs	0.89	86.7%	
	MI between-RSNs	0.95	89.5%	
	MI within-RSNs	0.78	80%	
**Scale 32**	*r* between-RSNs	0.98	96.2%	< 0.0001
	r within-RSNs	0.93	86.7%	
	MI between-RSNs	0.78	77.1%	
	MI within-RSNs	0.66	73.3%	
**Scale 64**	*r* between-RSNs	0.97	98.1%	< 0.0001
	r within-RSNs	0.93	86.7%	
	MI between-RSNs	0.78	78.1%	
	MI within-RSNs	0.72	80%	
**Scale 128**	*r* between-RSNs	0.98	98.1%	< 0.0001
	r within-RSNs	0.95	86.7%	
	MI between-RSNs	0.80	77.1%	
	MI within-RSNs	0.68	80%	

**Scale 256**	*r* between-RSNs	0.98	97.1%	< 0.0001
	r within-RSNs	0.95	86.7%	
	MI between-RSNs	0.83	76.2%	
	MI within-RSNs	0.71	80%	
**Scale 512**	*r* between-RSNs	0.98	98.1%	< 0.0001
	r within-RSNs	0.95	86.7%	
	MI between-RSNs	0.86	79%	
	MI within-RSNs	0.72	80%	

3. References

Bassett, D. S., Meyer-Lindenberg, A., Achard, S., Duke, T., and Bullmore, E. (2006). Adaptive reconfiguration of fractal small-world human brain functional networks. *Proc. Natl. Acad. Sci.* 103, 19518–19523. doi:10.1073/pnas.0606005103.

Jiang, A.-H., Huang, X.-C., Zhang, Z.-H., Li, J., Zhang, Z.-Y., and Hua, H.-X. (2010). Mutual information algorithms. *Mech. Syst. Signal Process.* 24, 2947–2960. doi:10.1016/j.ymssp.2010.05.015.

Shannon, C. E. (1948). A Mathematical Theory of Communication. *Bell Syst. Tech. J.* 27, 379–423. doi:10.1002/j.1538-7305.1948.tb01338.x.

Steuer, R., Kurths, J., Daub, C. O., Weise, J., and Selbig, J. (2002). The mutual information: Detecting and evaluating dependencies between variables. *Bioinformatics* 18, S231–S240. doi:10.1093/bioinformatics/18.suppl_2.S231.

van den Heuvel, M. P., and Fornito, A. (2014). Brain Networks in Schizophrenia. *Neuropsychol. Rev.* 24, 32–48. doi:10.1007/s11065-014-9248-7.

Yekutieli, D., and Benjamini, Y. (2001). under dependency. *Ann. Stat.* 29, 1165–1188. doi:10.1214/aos/1013699998.
